# An Eight-Year-Old Child With Sneddon Syndrome: A Rare Case Report

**DOI:** 10.7759/cureus.40054

**Published:** 2023-06-06

**Authors:** Maheshwari Nallur Siddaraju, Archana Samynathan, Sowjanya Kurakula, Priyadarshini Kharge, Sanjana D Nalla

**Affiliations:** 1 Dermatology, Bangalore Medical College and Research Institute, Bangalore, IND; 2 Dermatology, George Washington University, Virginia, USA; 3 Obstetrics and Gynecology, Mamta Institute of Medical Sciences, Khammam, IND; 4 Obstetrics and Gynecology, Sekgoma Memorial Hospital, Serowe, BWA; 5 Obstetrics and Gynecology, Nyangabgwe Referral Hospital, Francistown, BWA; 6 Obstetrics and Gynecology, Gandhi Medical College, Secunderabad, IND; 7 School of Medicine, Lake Erie College of Osteopathic Medicine, Erie, USA

**Keywords:** livedo racemosa, case report, systemic vasculitis, pediatric stroke, cerebrovascular accident, livedo reticularis

## Abstract

We present a rare case of slow-progressing neurocutaneous vasculopathy described as Sneddon syndrome. A child presented with global developmental delay, congenital livedo racemosa, unilateral vision loss, and a past history of focal neurological deficit. Our main objective is to make physicians aware of this nature of presentation in children.

## Introduction

The presentation of neurological symptoms and widespread livedo racemosa raise clinical suspicion of Sneddon syndrome. The exact causative factor is unknown. The mode of inheritance is often sporadic but autosomal dominance with variable penetrance is seen in rare familial cases. It is considered to be part of the clinical spectrum of primary antiphospholipid syndrome. Women of age between 20 and 42 are predominantly affected [1].

Apart from nervous system involvement, this syndrome also affects organs like the kidneys, heart, and eyes [2]. The mortality rate is estimated to be 9.5% according to a study [3]. Deficits in cognition, visual perception, and visuospatial skills are associated with a relatively poor neuropsychiatric prognosis of Sneddon syndrome [4].

## Case presentation

**Figure 1:**
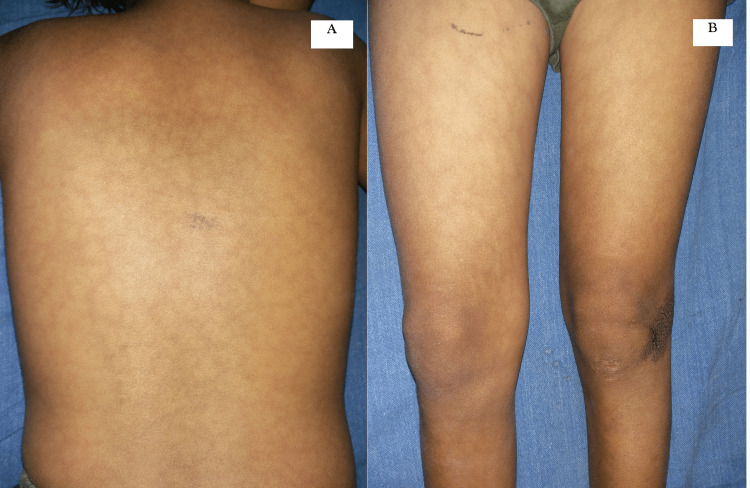
Livedo racemosa with discretely bluish to erythematous complete rings, closely arranged in a netlike pattern on the back (A) and lower extremities (B)

An eight-year-old girl presented with a history of congenital red-arborizing rash on the back and abdomen, gradually progressing to involve the rest of her body over five to six years (Figure 1).

The patient was born to second-degree consanguineous parents with uneventful antenatal and postnatal history. History was significant for delayed physical and mental development with no formal evaluation and work-up. Frequent episodes of headache, dizziness, weakness of the right side of the body, and loss of right eye vision at the age of seven were present. Over the course of one year, she had a retinal detachment in the right eye leading to phthisis bulbi, and the left eye showed changes of familial exudative vitreoretinopathy (FEVR), treated with laser photocoagulation. 

**Figure 2:**
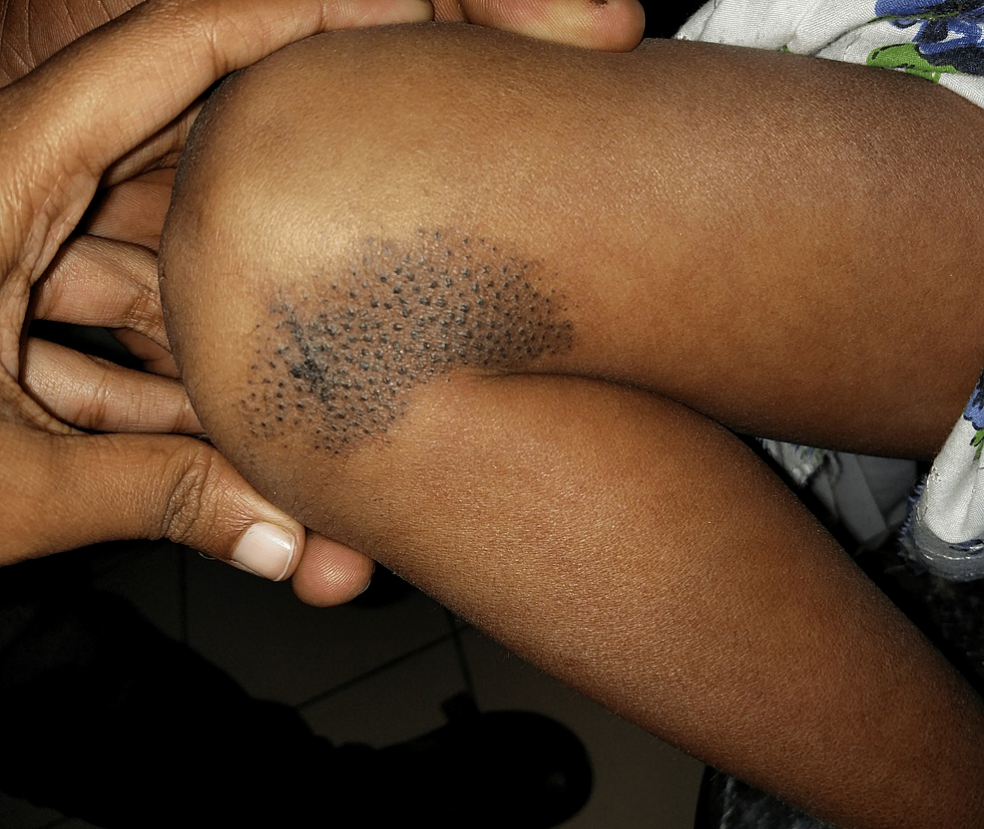
Nevus comedonicus seen as multiple grouped keratotic follicular plugs on the left knee

Cutaneous examination revealed an overlap of livedo racemosa and livedo reticularis, in the form of bluish to erythematous complete rings, closely arranged in a net-like pattern in a background of normal-looking skin all over the body including palms and soles with relatively sparing the central facial area. An isolated Nevus comedonicus was noticed over the left knee as multiple grouped keratotic follicular plugs (Figure 2).

The results of routine laboratory investigations, including complete blood count, comprehensive metabolic panel, and inflammatory markers, were within normal limits. Connective tissue disorders, including mixed connective tissue disorder, lupus, and Behcet's disease, were ruled out based on the negative anti-nuclear antibody, anti-U1 ribonucleoprotein, and anti-Sjogren's-syndrome-related antigen A autoantibody panel, and no association with human leukocyte antigen-B5. On neurological examination, the patient had decreased intelligence, loss of vision in the right eye, normal hearing and speech, sensory and motor functions, and normal tendon reflexes. A cerebral angiogram revealed bilateral internal carotid artery narrowing with collaterals from the external carotid artery and vertebral artery with bilateral segmental aneurysms suggesting vasculopathy. Magnetic resonance imaging of the brain showed gliosis representing vascular insults in the left frontoparietal and right frontal regions. Oral aspirin was initiated by the neurology team for stroke prophylaxis.

**Figure 3:**
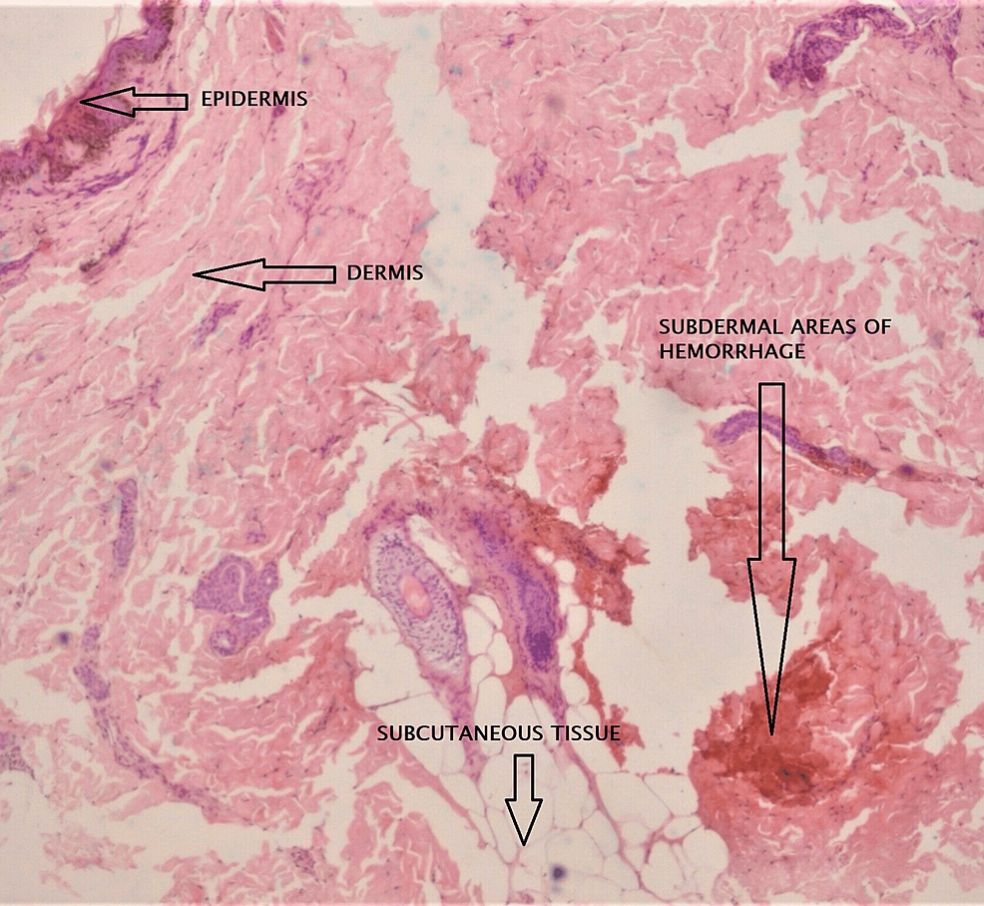
Histologic sample taken out from the border of livedo racemosa on the back showing thrombosis in medium and small-sized arteries and organizing hemorrhage at the junction of the dermis and subcutaneous tissue

Excisional skin biopsy from the central part of livedo racemosa was significant for thrombosis in medium and small-sized arteries and organizing hemorrhage at the junction of the dermis and subcutaneous tissue on histopathology, which translated to the clinical picture of the central pale area leading to the manifestation of a ringed net-like pattern (Figure 3).

With the cutaneous finding of livedo racemosa, histopathological features, thrombotic evidence in the computed tomography angiogram, and vasculopathy on magnetic resonance imaging, the patient was diagnosed to have Sneddon syndrome. This is one of the few cases reported of this syndrome manifesting in children at an early age.

## Discussion

Sneddon syndrome is a rare non-inflammatory disorder involving small and medium-sized arteries characterized by cerebrovascular thrombotic episodes and a striking cutaneous component of livedo racemosa [1]. The incidence of Sneddon syndrome is estimated to be four cases per million per year manifesting in the second to fourth decade of life with a female: male ratio of 2: 1. However, pediatric cases do exist [4].

Livedo racemosa is characterized by erythematous irregular, closely arranged rings and distributed most commonly over limbs, the trunk, buttocks, face, hands, and feet [5]. In most cases, skin lesions precede neurological symptoms similar to our case, but in a few, livedo racemosa has been noticed at the time of an episode of stroke [1].

The pattern of livedo racemosa is due to the stasis of blood in the superficial small to medium-sized arteries at the junction of the dermis and subcutaneous tissue either due to the hyperviscosity of the blood or due to the obstruction in the dermal arteries and capillaries [1,6]. The permanent impairment of blood flow is due to the proliferation and migration of tunica media smooth muscles, thus differentiating livedo racemosa from livedo reticularis caused by temporary vasoconstriction [1]. Livedo racemosa is a cutaneous feature in antiphospholipid antibody syndrome, systemic lupus erythematosus (with or without antiphospholipid), essential thrombocythemia, thromboangiitis obliterans, polycythemia vera, and polyarteritis nodosa, which are excluded by blood tests and histopathology in this patient [1,7]. The pathological cause of neurological symptoms is ischemic stroke presenting variably as hemiparesis, aphasia, sensory disturbances, visual field defects, and headache [8]. Due to multiple cerebral infarcts, patients may develop cognitive dysfunctions manifesting as disturbances in attention, memory, concentration, and depression [8]. Sneddon syndrome is thus classified based on the cause into idiopathic, primary antiphospholipid syndrome-associated, and systemic lupus erythematosus-associated [9].

Extracranial vascular events include deep venous thrombosis of the legs, pulmonary embolisms, and spontaneous abortions [5]. Cardiac complications include endocarditis, valvular regurgitation, and segmental wall motion abnormalities [5,6]. Known ophthalmologic complications are central retinal artery/vein occlusion, retinal neovascularization, visual field defects, and internuclear ophthalmoplegia [1]. Of significant mention is nevus comedonicus syndrome, wherein widespread nevus comedonicus lesions, congenital cataracts, microcephaly, and skeletal abnormalities are seen, and it was ruled out with the absence of other clinical features except for the isolated nevus comedonicus [10]. 

The management of Sneddon syndrome has no objective guidelines due to the rarity of the disease, which poses a challenge for clinical trials. The goal of the treatment is to prevent further ischemic insults in the skin, cerebral and extracerebral vasculature, and symptomatic control. Skin lesions associated with pain can be reduced by nifedipine [1]. Antiplatelet and oral anticoagulants remain the mainstay of treatment to prevent the occurrence of cerebrovascular complications [10]. Neurological symptoms are reported to be improved with intravenous cyclophosphamide [11]. The role, risks, and benefits of immunosuppressants and corticosteroids need validation and may prove detrimental considering the lifelong need for risk management. Further studies are necessary to explore treatment options in the pediatric age group. Considering the age, nature of symptoms, which were more neurological, and lack of skin pain or ulceration, our patient was treated with aspirin prophylaxis but was lost to follow-up.

## Conclusions

Sneddon syndrome preferentially involves cerebral and cutaneous small to medium-sized vasculature. Cutaneous findings of livedo racemosa in the setting of cerebral events, especially in younger females, warrant Sneddon syndrome in the list of differential diagnoses and as a diagnosis of exclusion. Optimal management remains unsolved. We would hereby emphasize considering Sneddon syndrome as a potential diagnosis in the presence of livedo racemosa and cerebral symptoms, irrespective of age, and the prompt initiation of antiplatelet and/or anticoagulative prophylaxis. The importance of follow-up in preventing irreversible complications and limiting disability due to neurological, cardiovascular, and ophthalmological complications cannot be overstated.
